# Interrelations between the main seed quality characteristics of narrowleaf lupine from the VIR collection

**DOI:** 10.18699/vjgb-25-05

**Published:** 2025-02

**Authors:** T.V. Shelenga, A.V. Salikova, V.S. Popov, G.P. Egorova, L.L. Malyshev, M.A. Vishnyakova

**Affiliations:** Federal Research Centre the N.I. Vavilov All-Russian Institute of Plant Genetic Resources (VIR), St. Petersburg, Russia; Federal Research Centre the N.I. Vavilov All-Russian Institute of Plant Genetic Resources (VIR), St. Petersburg, Russia; Federal Research Centre the N.I. Vavilov All-Russian Institute of Plant Genetic Resources (VIR), St. Petersburg, Russia; Federal Research Centre the N.I. Vavilov All-Russian Institute of Plant Genetic Resources (VIR), St. Petersburg, Russia; Federal Research Centre the N.I. Vavilov All-Russian Institute of Plant Genetic Resources (VIR), St. Petersburg, Russia; Federal Research Centre the N.I. Vavilov All-Russian Institute of Plant Genetic Resources (VIR), St. Petersburg, Russia

**Keywords:** narrowleaf lupine, alkaloids, protein, oil, seed moisture, weather conditions, люпин узколистный, алкалоиды, белок, масло, влажность семян, погодные условия

## Abstract

The widespread use of narrowleaf lupine (NLL, Lupinus angustifolius L.) as a feed and food crop requires source material for breeding cultivars with high-quality seeds. The priority criterion for attributing NLL cultivars to the feed or food category is the content of alkaloids. At the same time, equally important seed quality indicators are the protein and oil content, as well as moisture content, which determines the possibility of long-term storage of seeds. For the first time in Russian lupine science, an attempt was made to study the relationships between all the listed characteristics of narrowleaf lupine seeds under the conditions of Northwest Russia (Pushkin town). Sixty-two accessions from the VIR collection were studied in 2019, 2020 and 2022. The range of variability of the studied characteristics was 27.8–37.6 % for protein, 3.9–7.3 % for oil, 1.6–2017.4 mg/100 g of dry matter (D.M.) for alkaloids, and 6.4–7.3 % for moisture. A significant negative correlation between the oil and protein content (–0.33) was observed only in 2019. No significant correlations between the protein and alkaloid content were found in the studied sample. Significant negative relationships were identified between the content of oil and alkaloids only in 2019 and 2020 (–0.38 and –0.27, respectively). In 2022, no correlations were identified. Obviously, the identification of regularities in these correlations requires many years of research taking into account weather conditions. The influence of weather on the concentration of alkaloids in seeds has been proven. The average amount of alkaloids for the sample in 2019 was 504.2 ± 77.7 mg/100 g D.M., 263.7 ± 38.6 mg/100 g D.M. in 2020, and 319.8 ± 51.4 mg/100 g D.M. in 2022. It confirmed the data previously obtained by the authors that the content of alkaloids in seeds increases significantly along with the precipitation deficiency. The temperature regime during this research did not affect this indicator. An increased air temperature contributed to the accumulation of oil, and an increase in precipitation contributed to the accumulation of protein. The most stable indicator independent of environmental conditions was the seed moisture. Accessions with the optimal combination of the main biochemical parameters that determine seed quality have been identified for breeding narrowleaf lupine cultivars in the region in question for feed and food purposes, as well as for green manure.

## Abstract (ru)

Широкое использование люпина узколистного (ЛУ) в качестве кормовой и продовольственной культуры возможно при наличии сортов с высоким качеством семян. Приоритетным признаком для отнесения сортов ЛУ к этой категории считается низкое содержание алкалоидов. Однако не менее важные показатели качества семян люпина – содержание белка, масла и влажность. Впервые в отечественном люпиноведении предпринята попытка изучения взаимосвязей между показателями всех перечисленных ингредиентов семян ЛУ в условиях Северо-Запада РФ (г. Пушкин). Шестьдесят два образца из коллекции ВИР были изучены в 2019, 2020 и 2022 гг. Изменчивость содержания белка составила 27.8–37.6 %, масла – 3.9–7.3 %, алкалоидов – 1.6–2017.4 мг/100 г сухого вещества (с. в.), влажности – 6.4–7.3 %. Достоверная отрицательная корреляция между содержанием масла и белка (–0.33) наблюдалась только в 2019 г. Значимых корреляций между содержанием белка и алкалоидов в изученной выборке не обнаружено. Достоверная отрицательная связь между содержанием масла и суммой алкалоидов выявлена только в 2019 и 2020 гг. (–0.38 и –0.27 соответственно). В 2022 г. связь не обнаружена. Очевидно, что для выявления закономерностей, характеризующих эти связи, требуется проведение многолетних экспериментов с учетом погодных условий. Доказано влияние погоды на концентрацию алкалоидов в семенах. Средняя по выборке сумма алкалоидов в 2019 г. составила 504.2 ± 77.7 мг/100 г с. в., в 2020 г. – 263.7 ± 38.6 мг/100 г с. в. и в 2022 г. – 319.8 ± 51.4 мг/100 г с. в. Подтверждены данные, полученные авторами ранее: содержание алкалоидов в семенах значительно возрастает при дефиците осадков. Температурный режим не оказал влияния на этот показатель. Повышенная температура воздуха способствовала накоплению масла, а увеличение осадков – накоплению белка. Влажность семян оказалась наиболее стабильным показателем, не зависящим от условий среды. Выявлены образцы с оптимальным сочетанием основных биохимических показателей, определяющих качество семян, для региональной селекции сортов ЛУ кормового и продовольственного назначения, а также сидеральных сортов.

## Introduction

Narrowleaf lupine (NLL) Lupinus angustifolius L., a grain legume crop adapted to relatively northern regions, is a vital source of vegetable protein and amino acids, a universal ingredient in feeds for farm animals and a promising component of the human diet. The main limiting factor for the use of NLL for these purposes is the content of quinolizidine alkaloids (QA) in seeds and green matter, which impart bitterness to them. In the 1930s, after the discovery of low-alkaloid mutants (Sengbusch, 1931), the creation of low alkaloid cultivars began.

Both earlier in the USSR and currently in the Russian Federation, these cultivars were and are being created using accessions from the collection of the N.I. Vavilov All-Russian Institute of Plant Genetic Resources (VIR) characterized as sources of valuable traits. Currently, NLL breeding is developing quite actively in this country (Egorova et al., 2017). The State Register of Selection Achievements for 2023 includes 29 cultivars, all of which are domestically bred ones (State Register…, 2023). Breeding is carried out in two directions of cultivar use, i. e. as fodder and as green manure. For example, a low-alkaloid feed cv. ‘Belogorsky 310’ and a high-alkaloid cv. ‘Oligarkh’ for green manure have been created at the Leningrad Research Institute for Agriculture “Belogorka” jointly with the VIR researchers and using the source material from the VIR collection (Egorova et al., 2017).

According to the standards adopted in some European countries and in Australia, the alkaloid content, i. e. alkaloidity of seeds intended for food and feed purposes, should not exceed 0.02 % of their dry weight (D.M.) (Frick et al., 2017). In the Russian Federation, the permissible level of alkaloid content for fodder lupine cultivars is from 0.1 to 0.3 % D.M. (State Standard R 54632-2011), and, according to the existing technical specifications developed at the All-Russian Research Institute of Lupine, it is 0.04 % D.M. for food lupine (Specification No. 9716-004-00668502-2008). In everyday practice, the alkaloid seed content at the level of 0.05 % is considered a borderline value for distinguishing low-alkaloid and highalkaloid forms (Lee et al., 2007).

Along with the alkaloid content, the quality of NLL seeds is also determined by other metabolites, the main ones being protein and oil. The protein content in the seeds of narrowleaf lupine from the VIR collection was recorded at 34–36 % (Egorova et al., 2019). The oil content in the NLL collection accessions varies within 6.5–8.4 % (Benken et al., 1993).

High-protein cultivars with low alkaloid content are especially valuable because not only the grain, but also the green matter is eaten by all types of farm animals. Lupine is consumed freshly mown, in the form of compound feeds, silage, haylage, grain haylage, and bran (Kuptsov, Takunov, 2006).

Lupine oil is a functional ingredient and a valuable source of edible fats. The content of oleic, linoleic and linolenic fatty acids, of tocopherols, carotenoids, triglycerides, and triacylglycerols is at a high level. The oil of bitter cultivars contains a large number of phenolic compounds, although it has a lower antioxidant activity compared to that of sweet lupine oil (Siger et al., 2017).

Based on the above, evaluation of a lupine collection only by alkaloid content is not enough for recommending accessions as source material for creating feed and food cultivars. Accessions with a combination of high protein and oil and low alkaloid content are required. An important quality indicator of seeds is also their moisture content, which determines the suitability of seeds for storage and processing (State Standard R 52325-2005; Wang et al., 2001).

To which extent are the optimal values of these features compatible within one genotype, what are the regularity in relationships between them and their variability in the gene pool, and to which extent do they depend on growing conditions? Unfortunately, the answers to these questions in the world scientific literature are fragmentary and ambiguous. Therefore, the objectives of this article were to summarize the results of the biochemical analysis of a set of seed quality features, such as the content of protein, oil, alkaloids, and moisture in a sample of NLL accessions from the VIR collection grown for three years in Pushkin, Pushkinsky District of St. Petersburg (in the Northwest of the Russian Federation), to determine the degree of variability of the studied features, their dependency on weather factors in the region of research, and to identify the source material for breeding for high seed quality.

## Materials and methods

Plant materials and cultivation conditions. The object of the study was a sample of 62 NLL accessions from the VIR collection, selected according to the representativeness of their breeding status and alkaloidity. All the accessions were grown in 2019, 2020 and 2022 in the fields of the “Pushkin and Pavlovsk Laboratories” Scientific and Production Base of VIR (Pushkin, Pushkinsky District of St. Petersburg, 59°42′45.5ʺN 30°25′05.8ʺE) according to the methodology adopted for grain legumes (Vishniyakova et al., 2018). The growing area belongs to the Atlantic-continental region of the temperate climate zone.

Weather conditions during the years of research varied greatly (Supplementary Figure S1a, b)1. The coldest year was 2020, though the temperature in June of this year was slightly higher than the average long-term values. Average monthly air temperatures in June–August 2022 significantly exceeded the long-term average. Uneven precipitation was observed over the years of research. For example, the amount of precipitation in June 2019 was three times less than the average for this month in 2020 and 2022, and almost two times less in August of this year. The total precipitation in July–August 2020 and 2022 exceeded the long-term average. The greatest amount of precipitation over the years of research was recorded in August 2022 (Fig. S1a).


Supplementary Materials are available in the online version of the paper: https://vavilov.elpub.ru/jour/manager/files/Suppl_Shelenga_Engl_29_1.pdf


Methods. The alkaloid content was determined on an Agilent 6850 gas chromatograph coupled with an Agilent 5975 mass spectrometer (Agilent Technologies, USA) in extracts obtained by sequentially adding ethyl acetate and an aqueous solution of sodium hydroxide to samples of NLL flour. A caffeine solution was used as an internal standard (Kushnareva et al., 2020; Vishnyakova et al., 2023). The protein, oil and moisture content in the NLL seed flour samples were determined using the developed calibration models by near-infrared spectroscopy (NIRS) on a Matrix-1 IR analyzer (Bruker, Germany) (Popov et al., 2024).

To carry out statistical processing of the results obtained in 2019, 2020 and 2022, the samples of accessions were aligned to 40 units, the accessions were selected randomly. Statistical processing was performed using the Statistica 12.0 software package (StatSoft, Inc. (2019), www.statsoft.com). The statistical processing included calculation of the main descriptive statistics (mean, error of the mean, and coefficient of variation); analysis of variance for assessing the significance of differences between the accessions reproduced in different years with calculation of the least significant difference at p = 0.05; calculation of coefficients of rank correlation between the content of alkaloids and other biochemical indicators in different years of plant life to assess the stability of trait manifestation in accessions; factor analysis of the correlation system of biochemical traits

## Results

In addition to the previously obtained results on the alkaloid content in the seeds of the NLL sample studied in 2019–2020, the present study added an assessment of this trait in 2022 (Supplementary Table S1). As we showed earlier (Vishnyakova et al., 2023), this trait is highly weather-dependent, and the results of the third year of research make the picture more objective.

The qualitative composition of QA, characteristic of L. angustifolius and determined for the accessions in the studied sample earlier, is stable. The dominant alkaloid lupanine is followed in the descending order by 13-hydroxylupanine, sparteine, angustifoline and isolupanine (Kushnareva et al., 2020). The average long-term values (mg/100 g D.M.) for the entire research period were 252.9 for lupanine, 40.3 for 13-hydroxylupanine, 27.8 for sparteine, 4.6 for angustifoline, 2.9 for isolupanine, and 328.5 for the total alkaloids (Table S1). The variability (CV) of the alkaloid content exceeded 100 % (Table S2).

**Fig. 1.**
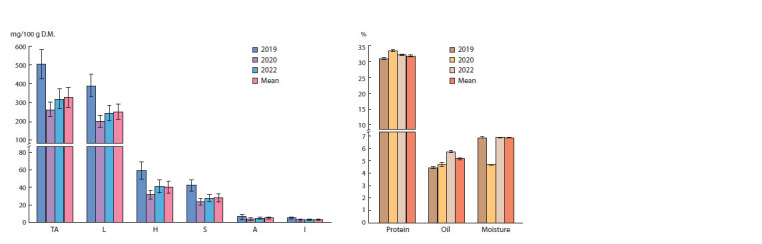
Total alkaloids and the content of (a) individual alkaloids; (b) protein, oil and moisture content in narrowleaf lupine seeds. Average, minimum, maximum values of indicators for 2019, 2020 and 2022 and average indicators for three years are presented. Pushkin, Pushkinsky District of St. Petersburg, 2019–2020 and 2022.

The average total alkaloids (mg/100 g D.M.) for the sample were maximum in 2019 at 504.2 ± 77.7, intermediate in 2022 at 319.8 ± 51.4, and minimum in 2020 at 263.7 ± 38.6 (Table S2). In 2019, the values of individual alkaloids and their total exceeded the average for three years, in 2020 and 2022 they were below the average long-term values. The maximum value of the trait 2017.4 mg/100 g D.M. was also noted in 2019, and the minimum of 1.6 mg/100 g D.M., in 2022. The coefficients of variation for the content of both individual alkaloids and their total were very high; the latter was from 112.4 to 124.6 (CV > 100 %, an abnormal range of values) (Table S2). This indicates a very large diversity of the studied accessions of the collection in terms of alkaloid content (Fig. 1a).

Fluctuations in the ranges of alkaloid content variability in NLL seeds over the years of research, taking into account the high CV, make it possible to assume a significant influence of weather conditions on these traits. However, the quantitative ratio of individual alkaloids remained unchanged during the years of research. The highest indicators during all three years of research were demonstrated by lupanine. Average values were typical for 13-hydroxylupanine and sparteine, and minimum ones, for angustiofoline and isolupanine

The content of protein, oil and moisture in seeds found in the studied sample averaged 31.9; 5.2 and 6.9 %, respectively over the entire period of research (Table S3). The protein and moisture indicators of lupine seeds depended little on weather conditions and were characterized by a low degree of variability (CV <10 %). The oil content in NLL seeds had a medium degree of variability (10 <CV ≈ 20 %). In 2019, the lowest values of protein and oil content were noted along with the highest content of alkaloids. In 2020, the minimum indicators for alkaloids, maximum for protein, and average for oil were recorded. In 2022, the intermediate values of alkaloids corresponded to the intermediate values of protein and maximum values of oil. No visible fluctuations in the moisture content of NLL seeds were detected during the period of research (Fig. 1b, Table S2).

An analysis of the obtained results showed that the seeds of different accessions obtained in 2019, 2020, and 2022 differ significantly in terms of lupanine, sparteine, total alkaloids, protein, and oil content. A difference close to significant was observed for the values of angustiofoline and isolupanine. The differences between the NLL accessions in terms of 13-hydroxylupanine and moisture content were not significant. The rank correspondence coefficient (Table S4) showed a reliable match between the alkaloidity of the NLL accessions grown in different years according to their alkaloid status (low- and high-alkaloid forms). Thus, it can be asserted that the content of individual alkaloids, as well as their total, are features, the order of magnitude of which is determined by the characteristics (genotype) of an accession, and the value of which is determined by the influence of abiotic factors.

The concentration of individual alkaloids and their total content in NLL seeds in 2019 was significantly higher than in 2020 and 2022 (Fig. 1a, Table S4). On the contrary, protein values were significantly higher in 2020, and the oil content in 2022 (Fig. 1b, Table S4). No significant differences in moisture content were found between NLL seeds obtained in different years (Fig. 1b, Table S4). It was found that the values for protein in 2020, and protein and oil in 2022 were higher than the average three-year ones, and those for oil in 2020, and protein and oil in 2019 were lower. The seed moisture content in all the years of research practically corresponded to the average long-term data.

In 2022, a close relationship between the values for individual alkaloids, and between those for individual alkaloids and their total was confirmed in all the years of study (r = 0.87 or more). This relationship was previously established for 2019 and 2020 (Vishnyakova et al., 2023). In 2019, a reliable negative relationship of medium strength was observed between the indicators for oil and alkaloids (from –0.35 to –0.42), and for oil and protein (–0.34). In 2020, the direction of the relationship between the content of oil and alkaloids, and that of oil and protein remained, but it became weaker; its reliability was confirmed only for lupanine, sparteine and the total alkaloids (the maximum absolute values of alkaloid concentration). In 2022, an inverse relationship close to reliable remained relevant only for lupanine (–0.23). There was a direct reliable correlation between seed moisture and sparteine in 2019, and between moisture, protein, and oil content in 2020 (0.27; 0.30; 0.29, respectively). In 2019, a reliable negative correlation was found between the content of moisture and lupanine, sparteine, and the total alkaloids (–0.29; 0.34; –0.29, respectively), and a negative correlation close to reliable between the content of moisture and 13-hydroxylupanine (–0.25). There were no significant correlations between protein content and alkaloid values (see the Table).

**Table 1.**
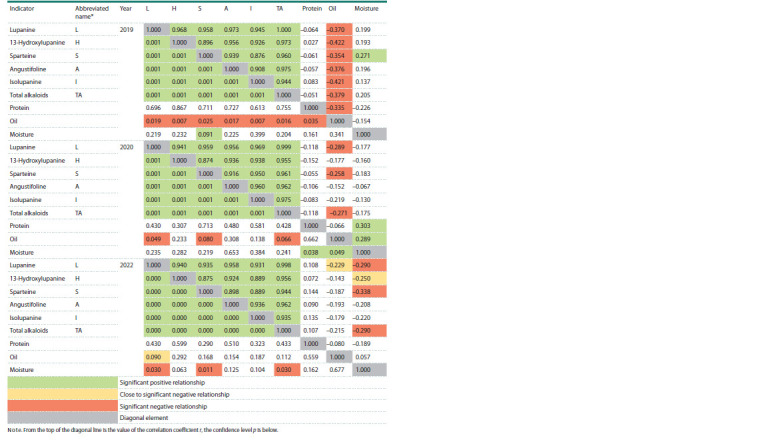
Correlation and factor structure of the variability of biochemical characteristics of seeds of narrowleaf lupine accessions in 2019, 2020, and 2022 Notе. From the top of the diagonal line is the value of the correlation coefficient r, the confidence level p is below

When analyzing the influence of weather conditions on the biochemical characteristics of NLL seeds, the previously established fact (Vishnyakova et al., 2023) that insufficient precipitation contributes to the accumulation of the main alkaloid lupanine and the total alkaloids was confirmed. These two indicators are related by a reliable negative correlation to the total amount of precipitation during the growing season (–0.996 and –0.997, respectively). A particularly noticeable lack of precipitation within three years was observed in June and August 2019, when 58 and 25 mm fell, respectively. The extremely low amount of precipitation for the region during this period (Fig. S1a) resulted in the maximum accumulation of lupanine and total alkaloids in 2019 (Fig. 1a, Table S2).

Higher air temperatures contributed to an increase in oil content, whereas an increase in precipitation contributed to the accumulation of protein in NLL seeds. Oil content and average air temperatures were linked by a reliable positive correlation (0.96), as were protein content and precipitation (0.96).

**Fig. 2.**
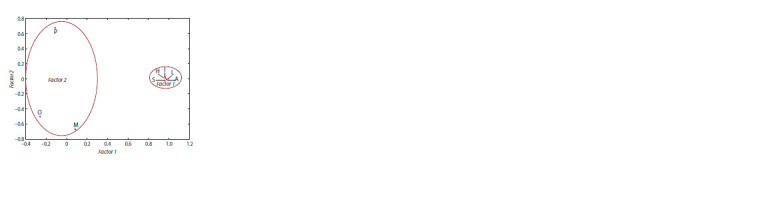
Factor structure of variability of average values of biochemical indicators determining the seed quality of narrowleaf lupine accessions in 2019, 2020, 2022 P – protein, O – oil, M – moisture, I – isolupanine, H – 13-hydroxylupanine, S – sparteine, L – lupanine, A – angustifoline.

The factor analysis of a correlation system allows a reduction of a high-dimensional feature space to a lower-dimensional one, in which the coordinate axes (factors) represent the center of concentration of features that correlate with each other. Factors are hidden variables that influence the observed features. Over three years of research, two factors describing a total of 77.7 % of the variability were identified in the variation structure. The first factor (Factor 1, 65.2 % of the variance) is associated with the variation of the indicators characterizing the content of alkaloids, while the second factor (Factor 2, 12.5 % of the variance) is negatively correlated with the protein content, and positively with the moisture and oil content in NLL seeds (Fig. 2). The alkaloid indicators (lupanine, 13-hydroxylupanine, sparteine, isolupanine, angustiofoline and their total content) are grouped in the right part of the graph. Most likely, this type of grouping is influenced by the fact that these compounds are linked by a single network of metabolic transformations. The indicators of protein, oil and moisture content in NLL seeds, concentrated in the left part of the figure, show a fairly large spread, which indicates the absence of a close relationship between them.

**Fig. 3.**
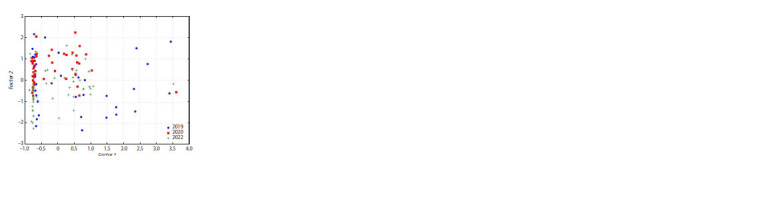
Position of narrowleaf lupine accessions in the space of selected factors (2019, 2020, 2022).

The eigenvalues of the factors calculated during the analysis for each NLL accession were used to describe the overall pattern of trait variability over the study period (Fig. 3). The distribution of accessions was influenced by the above-described factors. In 2019, a higher degree of dispersion was observed for both the first (Factor 1) and the second (Factor 2) factors. In 2020 and 2022, the spread of samples in the factor space decreased, they grouped mainly in its upper left, middle and partially in the lower left part, which is explained by a decrease in the alkaloid content, a smaller range of their variability and an increase in protein and oil content compared to 2019.

Since obtaining low-alkaloid forms has long been a priority direction of breeding, we paid special attention to the sources of this trait in the studied sample. These included 24 accessions with an alkaloid concentration in seeds of no more than 20–40 mg/100 g D.M. and 7 accessions with that of no more than 40 mg/100 g D.M. Of these accessions, 16 were characterized by a high protein content (above 30 %), 11 by a high oil content (above 5 %) (Table S1). The combination of a low alkaloid concentration and a high protein and oil content in one genotype characterizes them as sources of high-quality seeds for breeding NLL cultivars in the region for both fodder and food purposes. The group we have identified includes domestic and foreign NLL cultivars and lines: k-3172 (‘GL- 396’, Belarus), k-3329 (‘Line 7’, Russia), k-3502 (‘L-155’, Poland), k-3503 (‘Mutant 2’, Russia), k-3563 (‘Rommel’, South Africa), k-3627 (‘Dikaf-1’, Russia), k-3816 (‘Ladny 7’, Russia), as well as one wild-growing NLL accession k-3457 (‘GRC- 5008 A’, Greece)

The NLL accessions with the total alkaloid content of over 1,000 mg/100 g D.M. in seeds can be recommended as source material for creating green manure cultivars (Table S1). This group is mainly composed of foreign NLL cultivars and lines: k-96 (local, Ukraine), k-1526 (local, Ukraine), k-2183 (IGRIS, Poland), k-3562 (‘Slapska’, Czechoslovakia), k-3623 (18 86A250-2-4 EX LR2, Australia), and k-3814 (‘Oligarkh’, Russia).

The high-alkaloid cultivar ‘Oligarkh’ (k-3814) created at the Leningrad Research Institute for Agriculture “Belogorka” can be used both as raw material for the production of medicines and for creating new pharmacologically significant NLL cultivars. In our study, it demonstrated the following maximum values of individual alkaloid content (mg/100 g D.M.): 1487.3 for lupanine, 338.7 for 13-hydroxylupanine, 142.8 for sparteine, 38.6 for angustifoline, and 21.0 for isolupanine (Table S1). As is known, NLL can be a producer of alkaloids for the use in pharmacology and medicine (Vishnyakova et al., 2020).

## Discussion

The three-year data on the main biochemical characteristics that determine the quality of seeds in NLL accessions from the VIR collection grown in conditions of the Northwest of the Russian Federation (Leningrad Province) revealed significant variability in the alkaloid content. The data analysis shows that this is due to both genotypic determination and the influence of environmental conditions. The dependency of alkaloid content on weather factors during cultivation in these conditions was shown earlier (Vishnyakova et al., 2023). In our research, the protein content is characterized by low and oil content by medium variability. Seed moisture was the most stable characteristic. Seed moisture reaches about 15 % during harvesting, while before storing, its value should not exceed 10 % according to (State Standard R 52325-2005).

The statistical processing data confirmed that the alkaloid status (low and high alkaloid forms) was maintained by the NLL accessions reproduced in different years, i. e. the values of the individual alkaloids content, their total and ratio are determined by the genotype, while their variability is determined by the weather conditions of the year of reproduction. The previously established fact of an increase in the amount of alkaloids in seeds under dry conditions (Vishnyakova et al., 2023) was confirmed. A reliable negative correlation between the alkaloid content and the amount of precipitation was established

The influence of weather conditions on the alkaloid content in lupine seeds has been shown in different regions. In the Russian Federation, this was noted in the conditions of the Yaroslavl Province (Taran, Tsvik, 2017) and the Southwest zone of the Central Region (Ageeva, Pochutina, 2018). In Denmark, when three NLL cultivars were exposed to drought, the amount of alkaloids in the green matter at the stage of plant vegetation (before flowering) clearly increased, and then the genotypes responded differently to drought (Christiansen et al., 1997) by both increasing and decreasing the amount of alkaloids. Our opinion is that a comparison of these results can only be correct if the same plant organs (vegetative matter or seeds), the content of alkaloids in which is different, are assessed, and if the ontogenetic stages at which the study is conducted are the same

It is generally accepted that QA is synthesized in NLL in the chloroplasts of young leaves (Wink, 1991; 1993; Wink et al., 1995). The most intensive accumulation begins at the budding stage (Maknickienė, Asakavičiūtė, 2008). It has been shown that the expression of the identified to date seven candidate genes involved in the QA synthesis, and the alkaloids themselves, are detected in all plant tissues at the budding stage (Czepiel et al., 2021). However, the presence of alkaloids in plant tissues is detected as early as in young seedlings, into which the alkaloids pass from the germinating seeds (De Cortes Sánchez et al., 2021). That is, there is no unambiguous answer yet about the place and time of the onset of alkaloid synthesis in NLL. It is only obvious that the overwhelming fraction of QA is formed in green, above-ground tissues (Frick et al., 2017) with a small contribution from roots (Lee et al., 2007). By the time of beans formation, alkaloids enter the reproductive organs via the phloem (see a review by Vishnyakova, Krylova, 2022). It follows from this that the onset of the multi-stage synthesis of QAs in lupine and the time of their accumulation in seeds are separated in space and time. Numerous enzymes, transporters, and regulators are involved in this process. However, the expression of genes involved in the synthesis of alkaloids is no longer observed in mature seeds containing alkaloids (Czepiel et al., 2021). That is, only the accumulation process occurs in them. Therefore, it is obvious that the most vulnerable periods for the impact of stressors on the content of alkaloids are the synthesis and transport of the latter. The effect of early drought on a sharp increase in alkaloids in NLL has already been well proven (Frick et al., 2018). In the present research, the driest conditions in 2019 occurred in June, the time of budding (synthesis), and August (alkaloids delivery to the forming seeds). In general, the entire period of research has demonstrated a reliable negative relationship between the amount of precipitation during the entire vegetative period and the content of the main alkaloid lupanine, as well as the total alkaloids in NLL seeds (–0.996, –0.997, respectively)

An increase in alkaloid concentration under the influence of drought is used for their industrial production from producer plants, i. e. representatives of the genus Nicotiana, Papaver somniferum and Catharanthus roseus (Waller, Nowacki, 1978; Szabó et al., 2003; Jaleel et al., 2007; Amirjani, 2013). They are purposefully exposed to drought stress to increase the yield of alkaloids (Kleinwächter, Selmar, 2015).

It should be noted that the almost 2-fold average increase in alkaloid concentration recorded for the sample in 2019 compared to 2020 is characteristic only of high- and moderately alkaloid accessions. The accessions with the alkaloid content of less than 0.05 % showed a relatively little change in this indicator in all three years of study. The low-alkaloid accessions were stable in the manifestation of the trait and did not transform into high-alkaloid ones under the influence of weather conditions

The temperature factor did not affect the alkaloid content in NLL seeds in our research; at the same time, it was mentioned above that the elevated air temperature contributed to the accumulation of oil, and precipitation contributed to the accumulation of protein.

The studied sample demonstrated a reliable negative correlation between the protein and oil content (–0.34) only in 2019. A strong negative relationship (r = –0.96, p <0.01) between the content of these metabolites in seeds was shown in the work of Australian scientists who studied six NLL cultivars in 55 locations in Western Australia (Cowling, Tarr, 2004). However, a study of other lupine species from the VIR collection reproduced in the Northwest of the Russian Federation demonstrated a positive relationship between the protein and oil content (Egorova et al., 2019). It can be assumed that these relationships manifest themselves differently in different weather conditions

No significant correlations were found between the protein and alkaloid content in the sample studied in the present research. Meanwhile, among 126 samples of white lupine (L. albus L.) accessions from the collection at Pullman (Washington, USA), ranked into six classes by the degree of alkaloidity, a higher protein content was found in seeds from the group of high-alkaloid accessions (Staples et al., 2017). Reliable negative relationships between the oil and alkaloid content were found only in 2019 and 2020 (–0.38 and –0.27, respectively). In 2022, no relationships were found. We believe that the search for regularities in these relationships requires long-term research with weather conditions duly accounted for. The most stable indicator in our research, independent of environmental conditions, was seed moisture

## Conclusion

The relationships between the main NLL seed quality indicators (content of alkaloids, protein, oil, and moisture) and the influence of weather conditions on them were studied for the first time in the conditions of the Northwest of the Russian Federation in the Leningrad Province. The limits of variability of these traits are shown. The absence of significant correlations between the content of protein and oil, and protein and alkaloids was noted. Reliable negative relationships were found between the content of oil and alkaloids only in 2019 and 2020. It is obvious that identifying the regularities in these relationships requires long-term research with weather conditions duly accounted for. The influence of weather on the concentration of alkaloids in seeds has been proven, namely, its significant increase in dry conditions. The temperature regime during the present research did not affect this indicator. An increased air temperature contributed to the accumulation of oil, whereas precipitation contributed to the accumulation of protein. The studied sample was found to contain accessions that combine the necessary indicators of the main (protein and oil) and secondary metabolites (alkaloids) that determine the NLL seed quality for the use as source material when creating new regionally adapted cultivars for food, fodder, green manure and pharmaceutical purposes

## Conflict of interest

The authors declare no conflict of interest.
